# On the Validity of Using Increases in 5-Year Survival Rates to Measure Success in the Fight against Cancer

**DOI:** 10.1371/journal.pone.0083100

**Published:** 2014-07-23

**Authors:** Yosef E. Maruvka, Min Tang, Franziska Michor

**Affiliations:** 1 Department of Biostatistics and Computational Biology, Dana-Farber Cancer Institute, Boston, Massachusetts, United States of America; 2 Department of Biostatistics, Harvard School of Public Health, Boston, Massachusetts, United States of America; University of Rochester, United States of America

## Abstract

**Background:**

The 5-year survival rate of cancer patients is the most commonly used statistic to reflect improvements in the war against cancer. This idea, however, was refuted based on an analysis showing that changes in 5-year survival over time bear no relationship with changes in cancer mortality.

**Methods:**

Here we show that progress in the fight against cancer can be evaluated by analyzing the association between 5-year survival rates and mortality rates normalized by the incidence (mortality over incidence, MOI). Changes in mortality rates are caused by improved clinical management as well as changing incidence rates, and since the latter can mask the effects of the former, it can also mask the correlation between survival and mortality rates. However, MOI is a more robust quantity and reflects improvements in cancer outcomes by overcoming the masking effect of changing incidence rates. Using population-based statistics for the US and the European Nordic countries, we determined the association of changes in 5-year survival rates and MOI.

**Results:**

We observed a strong correlation between changes in 5-year survival rates of cancer patients and changes in the MOI for all the countries tested. This finding demonstrates that there is no reason to assume that the improvements in 5-year survival rates are artificial. We obtained consistent results when examining the subset of cancer types whose incidence did not increase, suggesting that over-diagnosis does not obscure the results.

**Conclusions:**

We have demonstrated, via the negative correlation between changes in 5-year survival rates and changes in MOI, that increases in 5-year survival rates reflect real improvements over time made in the clinical management of cancer. Furthermore, we found that increases in 5-year survival rates are not predominantly artificial byproducts of lead-time bias, as implied in the literature. The survival measure alone can therefore be used for a rough approximation of the amount of progress in the clinical management of cancer, but should ideally be used with other measures.

## Introduction

Improvements in the 5-year survival rates of cancer patients have long been cited as proof of the increasing effectiveness of anti-cancer treatment modalities both in scientific articles [1]–[4] and in media and policy documents [5]. The use of 5-year survival rates to reach this conclusion, however, was criticized in 1990 by Sondik [6], who argued that improvements in diagnostic abilities can contribute to increases in 5-year survival rates of cancer even in the absence of any progress towards more effective clinical interventions. Ten years ago, Welch, Schwartz and Woloshin [7] carefully investigated this criticism. The authors posited that improvements in cancer treatments should simultaneously increase survival rates and reduce mortality rates, thus leading to a correlation between the two, while improvements in survival rates due to artificial causes (e.g. due to earlier diagnoses) would not be related to decreased mortality. Indeed, they found no correlation between improving survival rates and decreasing mortality rates, which would seem to suggest that there have been no improvements in cancer management. On the other hand, if incidence rates were rising solely due to an increasing occurrence of cancer, then no correlation with survival rates should be observed in equilibrium. However, if the incidence was rising due to earlier diagnoses, then a correlation with rising survival rates should be detected; the latter was the case and a relationship was identified.

These results were interpreted as follows: “It has been reported that improvements in treatment results for patients with malignant disease represent spurious effects of diagnosis at an early stage” [8]. The insight that 5-year survival rates are a misleading measure of real improvement has since become canonical and is now included in standard textbooks on cancer biology [9]. Furthermore, these results also strongly call into question the efficiency of clinical interventions for cancer patients, since a lack of correlation between mortality rates and 5-year survival would seem to indicate that clinical management of cancer does not have any positive effects on patient survival.

We hypothesized that changes in the mortality rate itself does not fully capture improvements in cancer treatments and for this reason may not correlate with increasing survival rates. Importantly, even if cancer treatments were effective for an increasing number of patients, the mortality rate could still rise if the incidence of the given cancer type itself was increasing.

To illustrate this point, consider for example non-Hodgkin lymphoma. In the US, this disease had an age-adjusted incidence rate of 11 and a mortality rate of 5.6 per 100,000 people per year in 1975, and an incidence of 20 and a mortality rate of 7.9 per 100,000 per year in 2000 [10]. Although it seems that the outlook for patients diagnosed with this particular type of cancer deteriorated between 1975 and 2000, since the mortality rate rose by 2.3, clinical management of this cancer type actually improved dramatically, as the expected mortality rate in 2000 would have been 10.2. The expected mortality rate is determined by the fact that the incidence almost doubled and therefore, because the mortality rate is not reported as normalized by incidence, it should have almost doubled as well. The fact that the actual mortality rate only rose to 7.9 instead of the expected 10.2 signifies an improvement in the clinical management of the disease.

Based on these considerations, a more indicative measure of an improvement in cancer management should then be the change in mortality rate normalized by the change in the incidence rate (mortality over incidence, MOI). In the above example, rates of 5.6/11 in 1975 compared to 7.9/20 in 2000 reflect a 25% improvement in the overall clinical management of the disease. At the same time, the 5-year survival rate changed from 47% in 1975 to 70% in 2000. In this situation, the 5-year survival rate increased but does not correlate with the change in mortality; it does, however, correlate with the change in MOI. Despite the similarities between the fatality rate (the proportion of deaths within a designated population of cases) and the mortality over incidence, these measures are not identical; the fatality rate is obtained from a given case population, while mortality over incidence does not necessarily use the same population for the mortality and incidence. Nevertheless, due to the similarities between these two concepts, MOI can serve as an approximation for the fatality rate.

To investigate if the improvement in survival rates is related to improvements in the clinical management of cancer, we thus need to compare the change in survival to the change in MOI and not to the change in mortality rate alone. In cases in which the incidence remains unchanged, which is very uncommon, this analysis reduces to the currently used approach of correlating survival to mortality. Note that “improvements in clinical management” refers to any changes in the way cancer patients are dealt with clinically, including the administration of new drugs, the use of a more effective dosing strategy of existing drugs, or simply a cost reduction making previously available treatments more affordable and thus more widely used.

## Methods

### Data

The age-adjusted incidence rates, mortality rates and 5-year survival rates for US patients were obtained from the SEER database using SEERStat (www.seer.cancer.gov/seerstat). Throughout the paper, “survival” refers to relative but not overall survival. We obtained data of all cancer types in the SEER database and subdivided them into types according to available definitions. For example, the classification “leukemia” was subdivided into acute lymphocytic leukemia, chronic lymphocytic leukemia, other lymphocytic leukemia, acute myeloid leukemia, acute monocytic leukemia, chronic myeloid leukemia, and others. We did not combine the different subtypes as they display divergent biological and clinical characteristics [10] and display different incidence trends over time. Using the SEERStat program, we analyzed three groups: male patients, female patients, and a combined database including patients of both genders. The combined database is not independent from the other two but contains the largest collection of cancer types, and thus is useful for further analysis.

In addition, we obtained data on 36 cancer types from the NORDCAN website [11], which contain information about incidence, mortality and 5-year survival rates for the Nordic countries: Denmark, Finland, Norway and Sweden. We excluded data from Icelandic patients because of the small population size of this country, which causes too much noise for valid analysis The Nordic datasets are superior in comparison to the SEER USA datasets, as the latter do not provide comprehensive coverage of all US states.

We then calculated the change in incidence rates and change in 5-year survival between two chosen time points (referred to as the initial and the final time points). In order to reduce the amount of variation in the data, we calculated the average incidence and 5-year survival rates over the course of several years (see SI). For the Nordic countries, we used data averaged over the period of 1964–1967 as the initial time point, and data averaged over the period of 2000–2003 as the final time point. For data from US patients, we used 1973–1976 and 2000–2003 as the initial and the final time periods.

Since patients diagnosed with cancer in a given year die a few years later on average, the incidence and survival rates of a given year are associated with the mortality rate of a later year. We thus determined the association between the incidence rate in year x and the mortality rate in year x+a. We performed sensitivity analyses by choosing a wide range of a between 0 and 6 years and obtained consistent results. Here, we present the results for a  = 3: we calculated the changes in mortality rates during 1967–1970 and 2003–2006 for the Nordic countries, and during 1976–1979 and 2003–2006 for the US. Note that the relationship between the year of diagnosis and the mortality year should take into account the distribution of the death time. However, this would necessitate the use of more information that unfortunately is usually not available. Therefore, we used one gap time (which can be the mean, median or mode) as a rough approximation for the whole distribution.

### Measures

We calculated the change in the incidence, mortality, 5-year survival rates, and mortality over incidence (MOI) between the chosen two time points in each dataset. We then measured the correlation between the change in the 5-year survival rate and the change in incidence and mortality rates as was done by Welch et al. [7]. For each cancer type, the authors [7] calculated the change in mortality, incidence and 5-year survival rates and produced a figure showing the change in mortality versus the change in survival; each point represented a specific cancer type. They then calculated the correlation between the change in mortality and change in 5-year survival, and performed a similar calculation for the change in 5-year survival and the change in incidence. The mortality and incidence rates have different magnitudes for different types of cancers; therefore, in order to be able to compare frequent and rare cancer types, they measured the change in the rates over a certain time period as a percentage of the initial value, rather than as an absolute value. This percentage was then plotted against the absolute change in 5-year survival rates over the same time period. In addition, we calculated the change in MOI over the two chosen time points, which was then given as an absolute value (i.e. the mortality at the final time divided by the incidence at the final time, minus the mortality at the initial time divided by the incidence at the initial time) since the MOI itself is a fraction.

### Analysis

For each dataset, we investigated the correlation between changes in survival and the other three measures, as outlined above, using Pearson's and Spearman's correlations, and linear regression. In addition, we analyzed the correlation between the change in the incidence and the change in the mortality using the same approaches.

## Results

**Figure 1:**
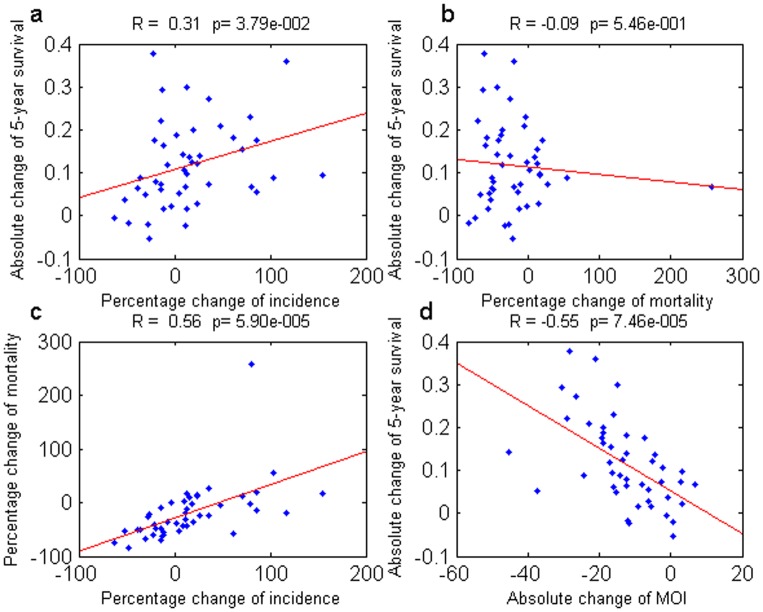
Correlations between different measures for the US dataset. (a) Change in 5-year survival versus change in mortality. (b) Change in 5-year survival versus change in incidence. (c) Change in mortality versus change in incidence. (d) Change in the 5-year survival versus change in mortality over incidence (MOI). The Pearson's correlation coefficient (R) and its p-value are displayed on top of each panel. The change in 5-year survival is strongly linearly associated with the change in MOI.

Using the datasets and analytic approaches outlined in Methods, we first investigated the correlation between changes in 5-year survival rates and incidence and mortality rates, as was done by Welch et al. [7]. The results for all datasets are summarized in **Table 1** **.** **Figure 1** displays the results for the US dataset as one particular example; all other plots are shown in **Figures S1–S11**. The correlation between the changes in 5-year survival rates and the changes in incidence and mortality rates are shown in **Figure 1a and b**. Similar to the findings by Welch et al [7], we also observed that there is no significant negative correlation between the change in survival and the change in mortality in all datasets. Meanwhile, in some datasets (see **Table 1**), there is a positive correlation between the change in survival and the change in incidence. These results were interpreted by Welch et al. [7] to indicate that improvements in survival rates are predominantly artificial, since a real improvement in the clinical management of cancer should simultaneously reduce mortality and enhance survival, while an artificial improvement, due to early diagnosis, increases survival rates and incidence rates without reducing the mortality. However, as shown in **Figure 1c**, there is a strong correlation between the change in incidence and the change in mortality. This finding indicates that much of the change in mortality is a result of the change in incidence, and therefore the correlation between the change in 5-year survival and the change in mortality due to improved treatment is obscured. In **Figure 1d**, we present the correlation between the change in 5-year survival rates and the change in MOI (Pearson's correlation coefficient −0.55, p-value  = 0.000075). In the SI, we show the correlation coefficients and p-values for alternative values of *a*. The correlation changes only slightly for these alternative intervals, and is significant for all the intervals investigated. The other datasets also display negative significant correlations, as is shown in the SI. The size of the correlation in some datasets is moderate (∼0.5), while it is strong in others. This difference may be due to different strengths of the artificial improvements in 5-year survival, but also may be due to the difference in data collection. Generally, the US datasets have weaker correlations than those from the Nordic countries, which may be due to the fact that with the US datasets, we divided cancer types into more detailed subtypes, which likely adds variation to the calculations. There is no general trend for the change in the correlation coefficient for different intervals that is shared by the different datasets that we used. See SI for a rigorous development of the negative correlation between the change in 5-year survival and MOI. We also repeated our analyses using Spearman's correlation (see SI), and found that the changes when using this metric were minor.

**Table 1:** Pearson's correlation coefficients for different pairs of measures.

Country	Gender	d(I) vs d(M)		d(I) vs d(S)		d(M) vs d(S)		d(S) vs d(M/I)	
		All types	d(I)<0	All types	d(I)<0	All types	d(I)<0	All types	d(I)<0
Denmark	Female	0.81++	0.13	0.01	0.45	−0.37*	−0.27	−0.69+	−0.69**
Denmark	Male	0.77++	0.19	0.14	0.5	−0.26	−0.43	−0.73++	−0.85***
Finland	Female	0.6***	0.85**	0.37*	0.01	−0.16	−0.4	−0.88++	−0.89***
Finland	Male	0.63***	0.62	0.43*	0.34	−0.06	−0.27	−0.81++	−0.87**
Norway	Female	0.75++	0.37	−0.06	0.51	−0.15	−0.2	−0.59***	−0.71
Norway	Male	0.45*	0.46	0.34	0.8	−0.05	0.03	−0.61***	−0.92*
Sweden	Female	0.71+	0.47	−0.05	0.1	−0.23	−0.61*	−0.54**	−0.75**
Sweden	Male	0.5**	0.32	0.45*	0.22	−0.05	−0.51	−0.59***	−0.62*
United States	Both genders	0.56+	0.56+	0.3*	0.3*	−0.09	−0.09	−0.55+	−0.55+
	Male	0.71++	0.66*	0.29	0.4	−0.008	−0.1	−0.4*	−0.77**
	Female	0.66+	0.19	0.16	0.1	−0.18	−0.63*	−0.55**	−0.56*

For each pair, the first column provides the Pearson correlation coefficient calculated by using all cancer types, and the second is calculated by using only cancer types whose incidence did not increase during the time period of observation. The quantity d(I) denotes change of incidence rates; d(M) the change of mortality rates; d(S) the change of 5-year survival rates; and d(M/I) the change of mortality over incidence rates, i.e. change of MOI. Significant p-values are indicated by * for P<0.05, ** for P<0.01, *** for P<0.001, + for P<0.0001 and ++ for P<0.00001.

Note that we have observed the pattern expected under real improvements in the clinical management of cancer; thus, there is no reason to assume that improvements in cancer treatment are not real, as implied by Welch et al. [7]. However, this observation in itself is not proof that the improvements in cancer treatment are real, because over-diagnosis [12] — the identification of cancer cases due to improved diagnostic tools that would not have resulted in death even without the diagnosis — can artificially create a negative correlation between the change in 5-year survival and the change in MOI. This fact arises because over-diagnosis artificially increases both survival and incidence, resulting in a negative correlation between the rates of survival and MOI even without any underlying improvements in the clinical management of cancer patients. It has recently become apparent that over-diagnosis is a problem when investigating incidence and prevalence rates of some cancer types [13]. In order to tackle the problem of over-diagnosis, we repeated our analyses of the correlation between the change in survival and the change in MOI using only those cancer types whose incidence did not increase during the chosen time period. We hypothesized that for those cancer types with stable or decreasing incidence rates, the contribution of over-diagnosis should be very low. For the US cohort, this approach led to the use of 35 cancer types.

When performing these analyses, we obtained a significant negative slope between changes in survival and MOI for the US male and combined datasets. For the US female dataset, we obtained a negative slope for all cases, but the slope was significant only when using mortality data from a time period of at least three years after the date at which the incidence was determined. These results are presented in the SI. These findings demonstrate that also for cancer types with almost no over-diagnosis, the negative correlation between changes in 5-year survival and in MOI still exist; thus, it is likely that the improvements in the 5-year survival rates are real even in cancer types where incidence rates did increase, as there is no reason to posit a meaningfully higher over-diagnosis rate in those cases.

The results for the US cohort are a typical example, and the figures for all other cohorts are presented in the SI. **Table 1** displays the correlation coefficients and their p-values of the following comparisons for all available datasets: changes in 5-year survival rates and incidence, changes in 5-year survival rates and mortality, changes in mortality and incidence, and changes in 5-year survival rates and MOI. Note that in all cases, there is a negative correlation between the rates of survival and MOI, which is always statistically significant. Also, by using only cancer types whose incidence did not increase in the Nordic countries, we consistently obtained a significantly negative slope for all datasets, except for the female population in Norway for which there were only 6 cancer types, a sample set potentially too small to allow for significant results. Note that the mortality and incidence rates are age-adjusted, and the adjustments of the US and the Nordic data were potentially done differently; this might thus represent the source for some of the differences we observed between the US and Nordic countries.

For the US population, we repeated all calculations using 10-year survival data instead of 5-year survival data and obtained consistent results. The correlation between the change in 10-year survival and the change in MOI was negative and significant for all patients as well as for both genders. Also, when we used only the cancer types for which the incidence did not increase, we obtained a significantly negative correlation (see SI).

## Discussion

In this paper, we have proposed an alternative approach to investigate the effectiveness of clinical interventions against cancer using information of incidence, survival, and mortality of cancer patients. We showed that by comparing survival to MOI (mortality normalized by incidence), the expected correlation patterns between the changes in 5-year survival and MOI are obtained. By doing so, we negated the previous claim by Welch et al. [7] that the expected correlation cannot be observed and therefore, that 5-year survival rates cannot be used to evaluate progress in the fight against cancer. In addition, we demonstrated that early diagnosis is not a major problem for the analysis of changes in survival rates. However, this finding by itself does not prove that the effects of artificial improvements in the 5-year survival are negligible; rather, it shows that there is no reason to assume that their effect is large, because even though MOI cannot improve artificially due to lead time bias, it can still artificially improve due to over-diagnosis.

To tackle the problem of over-diagnosis, we analyzed only those cancer types whose incidence did not increase during the time period of observation; thus there is no reason to believe that these cancer types are significantly influenced by over-diagnosis. For these cancer types, we also obtained a significantly negative correlation between the change in 5-year survival and the change in MOI. Therefore, for these cancer types, the improvement in 5-year survival rates is not due to early diagnosis or over-diagnosis. The fact that we obtained the same results for cancer types with an incidence that did not increase indicates that over-diagnosis does not play a major role in artificially enhancing the 5-year survival for any cancer type.

We found that in general, the changes in 5-year survival rates contain information that can be harvested to measure our progress in the war against cancer. While early diagnoses artificially increase the rates of 5-year survival, in general, their effect is minor relative to the effect of improvements in cancer treatments. This finding becomes apparent when the survival rates are compared to the mortality rates normalized by the incidence (MOI).

The survival measure and the MOI measure should ideally be used in tandem to assess improvements in cancer treatments, as no measure is perfect. Survival alone may include minor effects of lead-time bias. The MOI measure alone does not properly capture improved survival times in cases when mortality rates do not change. However, improvements in the survival measure alone can be used to provide a reliable indication of progress.

Note that the correlation between MOI and 5-year survival was already observed by Vostakolaei et al. [14] who showed that MOI was generally a good proxy for 5-year survival. However, our work differs from their investigation in that we have analyzed the correlation between the *change* in MOI and the *change* in 5-year survival, in order to determine whether improvements in survival rates are real. This question was not answered previously since the authors did not compare changes in those measurements over time, but rather looked at a single snapshot in time.

It is worth noting that other approaches to assessing the improvements in cancer treatment can be taken, such as analyzing the changes in the number and ratio of patients diagnosed at different stages of cancer development. However, such methods require more detailed information about the diagnostic tools used, and should be applied to specific types of cancers separately, thus differing from the wide-scale approach we applied here.

## Supporting Information

Figure S1 **The correlation between the different measures for the female cohort from Denmark.** (a) Change in mortality vs change in incidence. (b) Change in 5-year survival vs change in incidence. (c) Change in 5-year survival vs change in mortality. (d) Change in 5-year survival vs change in mortality over incidence (MOI). Pearson's correlation coefficient and its p-value are displayed on top of each panel. The change in 5-year survival is strongly linearly associated with the change in MOI. (e–h) Same as in (a–d), but including only those cancer types whose incidence decreased during the time of observation.
(BMP)

Figure S2 **The correlation between the different measures for the male cohort from Denmark.** (a) Change in mortality vs change in incidence. (b) Change in 5-year survival vs change in incidence. (c) Change in 5-year survival vs change in mortality. (d) Change in 5-year survival vs change in mortality over incidence (MOI). Pearson's correlation coefficient and its p-value are displayed on top of each panel. The change in 5-year survival is strongly linearly associated with the change in MOI. (e–h) Same as in (a–d), but including only those cancer types whose incidence decreased during the time of observation.
(BMP)

Figure S3 **The correlation between the different measures for the female cohort from Finland.** (a) Change in mortality vs change in incidence. (b) Change in 5-year survival vs change in incidence. (c) Change in 5-year survival vs change in mortality. (d) Change in 5-year survival vs change in mortality over incidence (MOI). Pearson's correlation coefficient and its p-value are displayed on top of each panel. The change in 5-year survival is strongly linearly associated with the change in MOI. (e–h) Same as in (a–d), but including only those cancer types whose incidence decreased during the time of observation.
(BMP)

Figure S4 **The correlation between the different measures for the male cohort from Finland.** (a) Change in mortality vs change in incidence. (b) Change in 5-year survival vs change in incidence. (c) Change in 5-year survival vs change in mortality. (d) Change in 5-year survival vs change in mortality over incidence (MOI). Pearson's correlation coefficient and its p-value are displayed on top of each panel. The change in 5-year survival is strongly linearly associated with the change in MOI. (e–h) Same as in (a–d), but including only those cancer types whose incidence decreased during the time of observation.
(BMP)

Figure S5 **The correlation between the different measures for the female cohort from Norway.** (a) Change in mortality vs change in incidence. (b) Change in 5-year survival vs change in incidence. (c) Change in 5-year survival vs change in mortality. (d) Change in 5-year survival vs change in mortality over incidence (MOI). Pearson's correlation coefficient and its p-value are displayed on top of each panel. The change in 5-year survival is strongly linearly associated with the change in MOI. (e–h) Same as in (a–d), but including only those cancer types whose incidence decreased during the time of observation.
(BMP)

Figure S6 **The correlation between the different measures for the male cohort from Norway.** (a) Change in mortality vs change in incidence. (b) Change in 5-year survival vs change in incidence. (c) Change in 5-year survival vs change in mortality. (d) Change in 5-year survival vs change in mortality over incidence (MOI). Pearson's correlation coefficient and its p-value are displayed on top of each panel. The change in 5-year survival is strongly linearly associated with the change in MOI. (e–h) Same as in (a–d), but including only those cancer types whose incidence decreased during the time of observation.
(BMP)

Figure S7 **The correlation between the different measures for the female cohort from Sweden.** (a) Change in mortality vs change in incidence. (b) Change in 5-year survival vs change in incidence. (c) Change in 5-year survival vs change in mortality. (d) Change in 5-year survival vs change in mortality over incidence (MOI). Pearson's correlation coefficient and its p-value are displayed on top of each panel. The change in 5-year survival is strongly linearly associated with the change in MOI. (e–h) Same as in (a–d), but including only those cancer types whose incidence decreased during the time of observation.
(BMP)

Figure S8 **The correlation between the different measures for the male cohort from Sweden.** (a) Change in mortality vs change in incidence. (b) Change in 5-year survival vs change in incidence. (c) Change in 5-year survival vs change in mortality. (d) Change in 5-year survival vs change in mortality over incidence (MOI). Pearson's correlation coefficient and its p-value are displayed on top of each panel. The change in 5-year survival is strongly linearly associated with the change in MOI. (e–h) Same as in (a–d), but including only those cancer types whose incidence decreased during the time of observation.
(BMP)

Figure S9 **The correlation between the different measures for both genders from the US.** (a) Change in mortality vs change in incidence. (b) Change in 5-year survival vs change in incidence. (c) Change in 5-year survival vs change in mortality. (d) Change in 5-year survival vs change in mortality over incidence (MOI). Pearson's correlation coefficient and its p-value are displayed on top of each panel. The change in 5-year survival is strongly linearly associated with the change in MOI. (e–h) Same as in (a–d), but including only those cancer types whose incidence decreased during the time of observation.
(BMP)

Figure S10 **The correlation between the different measures for the male US cohort.** (a) Change in mortality vs change in incidence. (b) Change in 5-year survival vs change in incidence. (c) Change in 5-year survival vs change in mortality. (d) Change in 5-year survival vs change in mortality over incidence (MOI). Pearson's correlation coefficient and its p-value are displayed on top of each panel. The change in 5-year survival is strongly linearly associated with the change in MOI. (e–h) Same as in (a–d), but including only those cancer types whose incidence decreased during the time of observation.
(BMP)

Figure S11 **The correlation between the different measures for the female US cohort.** (a) Change in mortality vs change in incidence. (b) Change in 5-year survival vs change in incidence. (c) Change in 5-year survival vs change in mortality. (d) Change in 5-year survival vs change in mortality over incidence (MOI). Pearson's correlation coefficient and its p-value are displayed on top of each panel. The change in 5-year survival is strongly linearly associated with the change in MOI. (e–h) Same as in (a–d), but including only those cancer types whose incidence decreased during the time of observation.
(BMP)

Table S1 **USA incidence and mortality rates.**
(XLSX)

Table S2 **USA survival rates.**
(XLSX)

Table S3 **European Nordic countries incidence.**
(XLS)

Table S4 **European Nordic countries mortality.**
(XLSX)

Table S5 **European Nordic countries survival rates.**
(XLSX)

Table S6 **Results. Both Pearson and spearman coefficients for all the estimated correlations is given.** This information was used to generate figure 1 and S1-S11.
(XLSX)
