# Supplementary figures and images for: On the Validity of Using Increases in 5-Year Survival Rates to Measure Success in the Fight against Cancer
Source: PLoS One. 2014 Jul 23;9(7):e83100. doi: 10.1371/journal.pone.0083100 (PMC4108307; doi:10.1371/journal.pone.0083100)

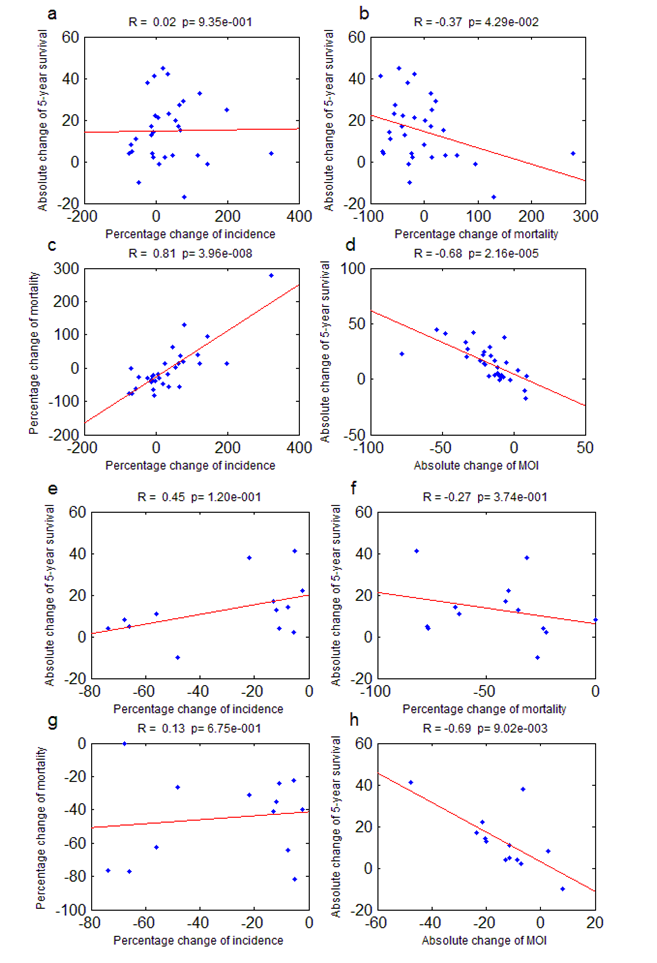

Supplement: Figure S1 — The correlation between the different measures for the female cohort from Denmark. (a) Change in mortality vs change in incidence. (b) Change in 5-year survival vs change in incidence. (c) Change in 5-year survival vs change in mortality. (d) Change in 5-year survival vs change in mortality over incidence (MOI). Pearson's correlation coefficient and its p-value are displayed on top of each panel. The change in 5-year survival is strongly linearly associated with the change in MOI. (e–h) Same as in (a–d), but including only those cancer types whose incidence decreased during the time of observation. (BMP) [file pone.0083100.s001.bmp]

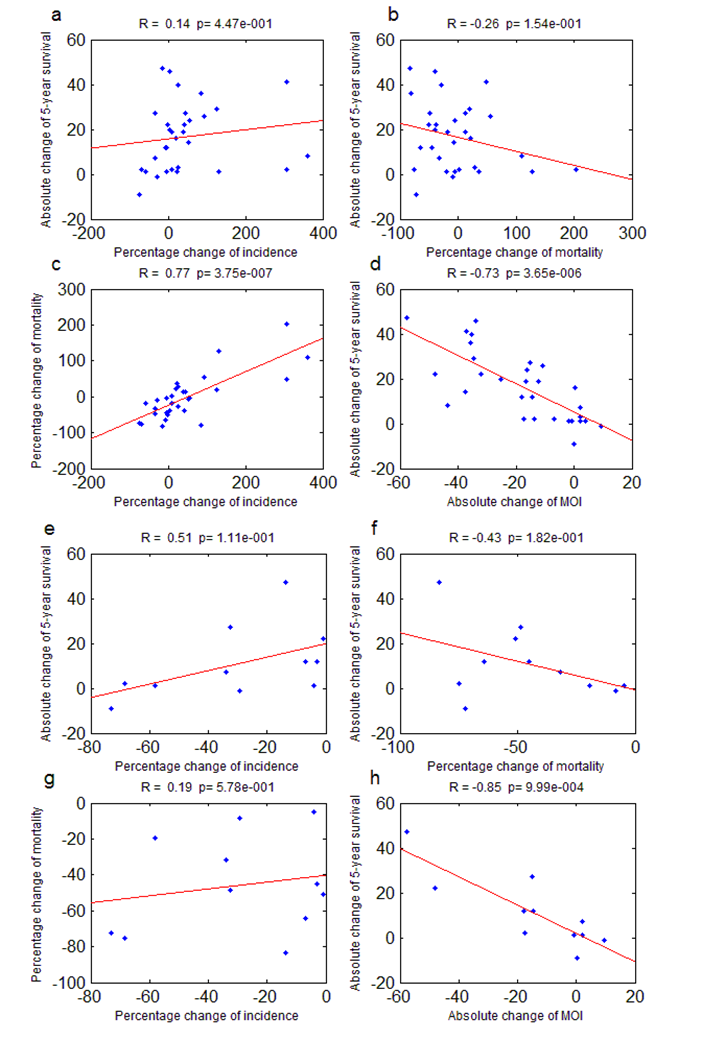

Supplement: Figure S2 — The correlation between the different measures for the male cohort from Denmark. (a) Change in mortality vs change in incidence. (b) Change in 5-year survival vs change in incidence. (c) Change in 5-year survival vs change in mortality. (d) Change in 5-year survival vs change in mortality over incidence (MOI). Pearson's correlation coefficient and its p-value are displayed on top of each panel. The change in 5-year survival is strongly linearly associated with the change in MOI. (e–h) Same as in (a–d), but including only those cancer types whose incidence decreased during the time of observation. (BMP) [file pone.0083100.s002.bmp]

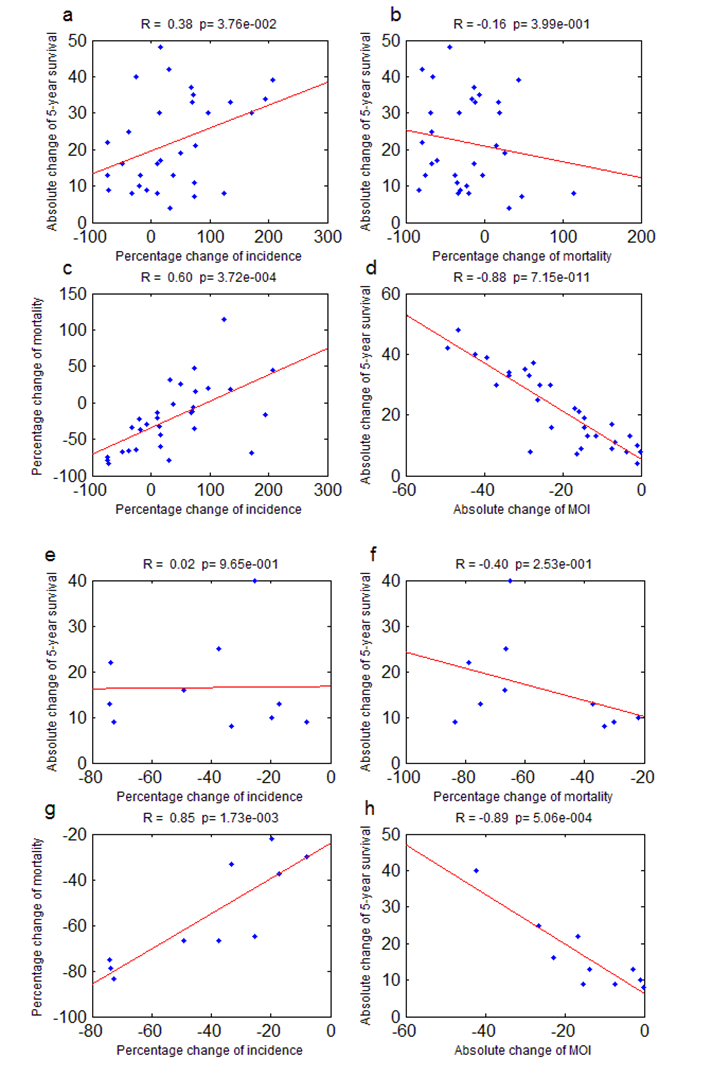

Supplement: Figure S3 — The correlation between the different measures for the female cohort from Finland. (a) Change in mortality vs change in incidence. (b) Change in 5-year survival vs change in incidence. (c) Change in 5-year survival vs change in mortality. (d) Change in 5-year survival vs change in mortality over incidence (MOI). Pearson's correlation coefficient and its p-value are displayed on top of each panel. The change in 5-year survival is strongly linearly associated with the change in MOI. (e–h) Same as in (a–d), but including only those cancer types whose incidence decreased during the time of observation. (BMP) [file pone.0083100.s003.bmp]

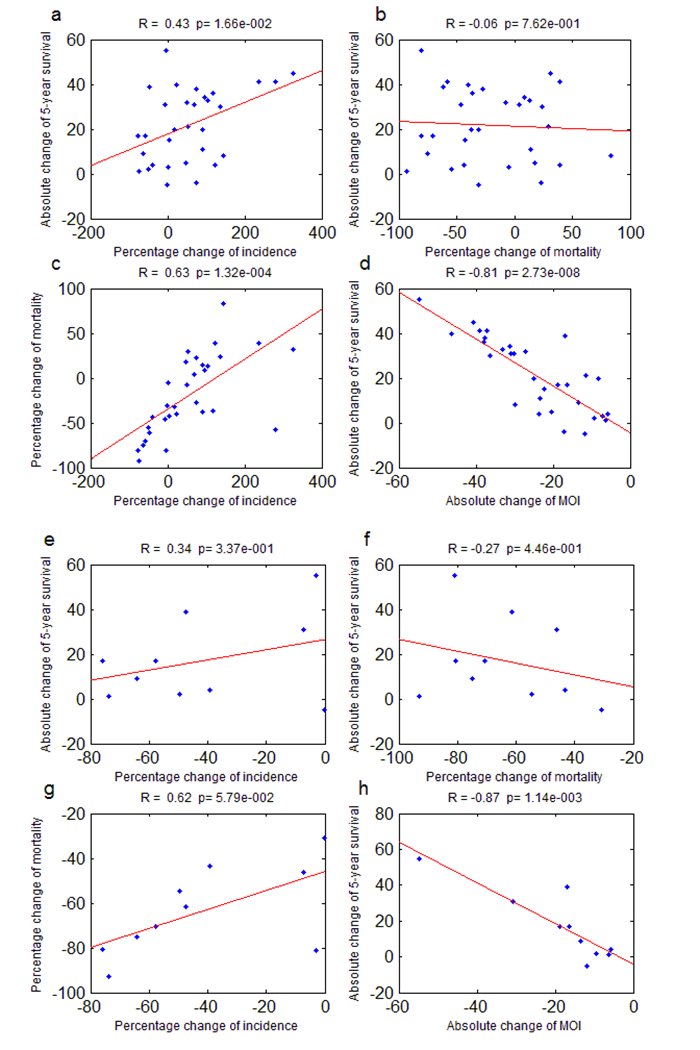

Supplement: Figure S4 — The correlation between the different measures for the male cohort from Finland. (a) Change in mortality vs change in incidence. (b) Change in 5-year survival vs change in incidence. (c) Change in 5-year survival vs change in mortality. (d) Change in 5-year survival vs change in mortality over incidence (MOI). Pearson's correlation coefficient and its p-value are displayed on top of each panel. The change in 5-year survival is strongly linearly associated with the change in MOI. (e–h) Same as in (a–d), but including only those cancer types whose incidence decreased during the time of observation. (BMP) [file pone.0083100.s004.bmp]

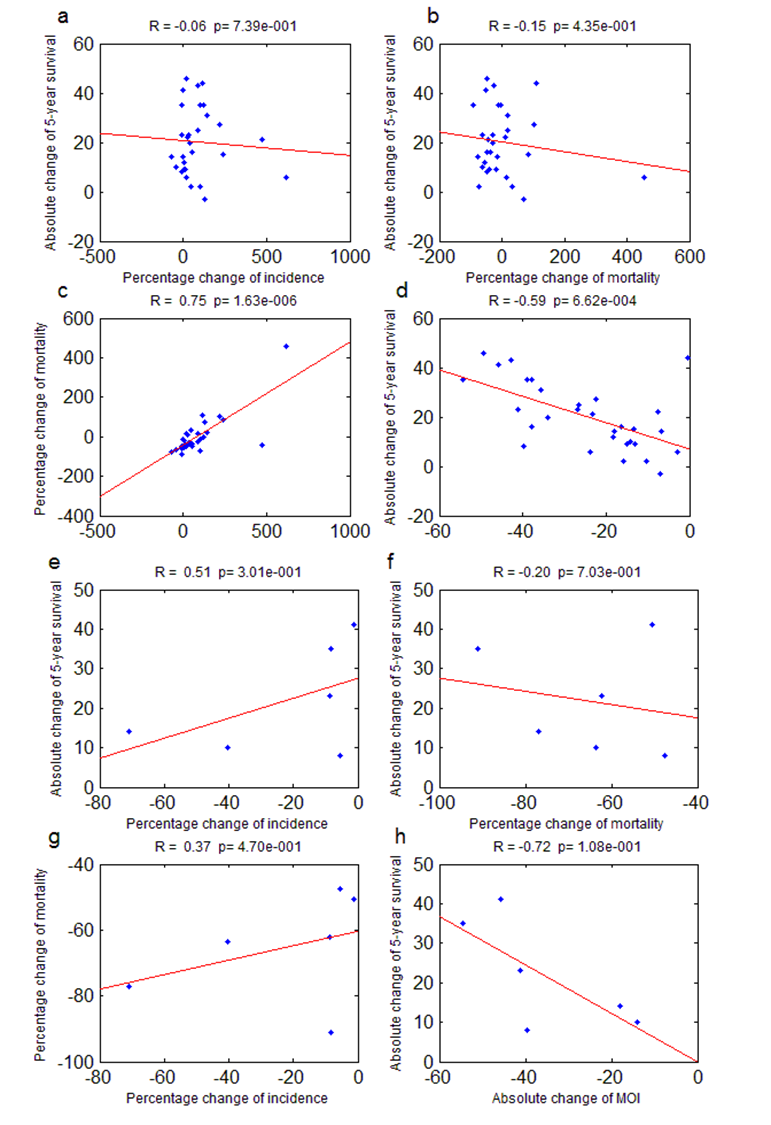

Supplement: Figure S5 — The correlation between the different measures for the female cohort from Norway. (a) Change in mortality vs change in incidence. (b) Change in 5-year survival vs change in incidence. (c) Change in 5-year survival vs change in mortality. (d) Change in 5-year survival vs change in mortality over incidence (MOI). Pearson's correlation coefficient and its p-value are displayed on top of each panel. The change in 5-year survival is strongly linearly associated with the change in MOI. (e–h) Same as in (a–d), but including only those cancer types whose incidence decreased during the time of observation. (BMP) [file pone.0083100.s005.bmp]

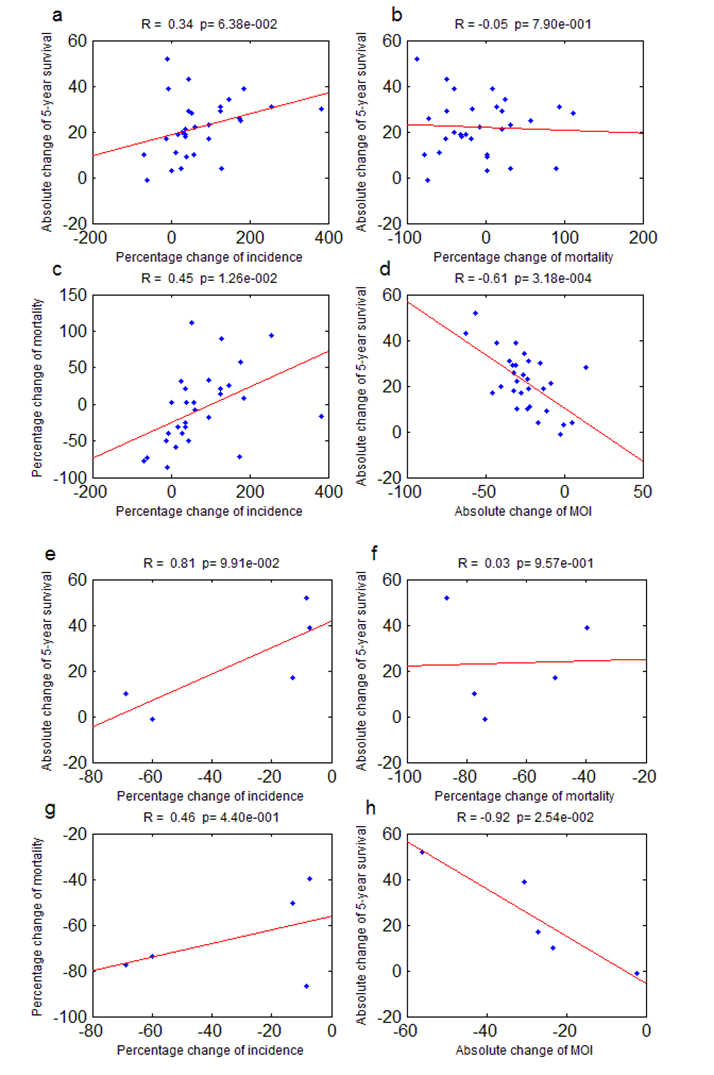

Supplement: Figure S6 — The correlation between the different measures for the male cohort from Norway. (a) Change in mortality vs change in incidence. (b) Change in 5-year survival vs change in incidence. (c) Change in 5-year survival vs change in mortality. (d) Change in 5-year survival vs change in mortality over incidence (MOI). Pearson's correlation coefficient and its p-value are displayed on top of each panel. The change in 5-year survival is strongly linearly associated with the change in MOI. (e–h) Same as in (a–d), but including only those cancer types whose incidence decreased during the time of observation. (BMP) [file pone.0083100.s006.bmp]

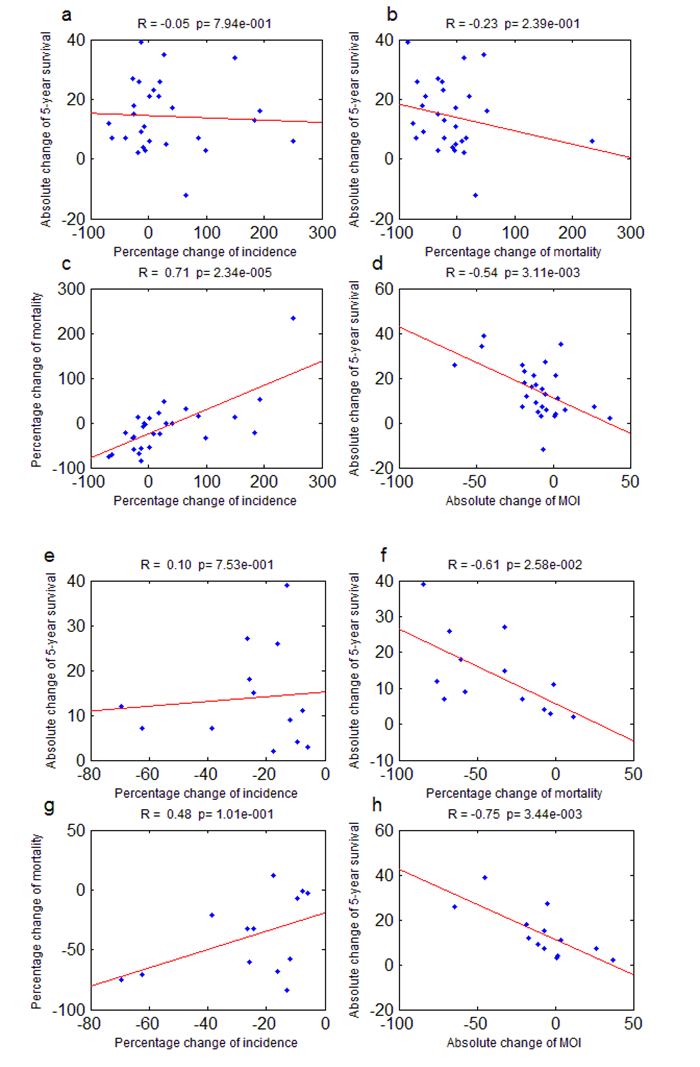

Supplement: Figure S7 — The correlation between the different measures for the female cohort from Sweden. (a) Change in mortality vs change in incidence. (b) Change in 5-year survival vs change in incidence. (c) Change in 5-year survival vs change in mortality. (d) Change in 5-year survival vs change in mortality over incidence (MOI). Pearson's correlation coefficient and its p-value are displayed on top of each panel. The change in 5-year survival is strongly linearly associated with the change in MOI. (e–h) Same as in (a–d), but including only those cancer types whose incidence decreased during the time of observation. (BMP) [file pone.0083100.s007.bmp]

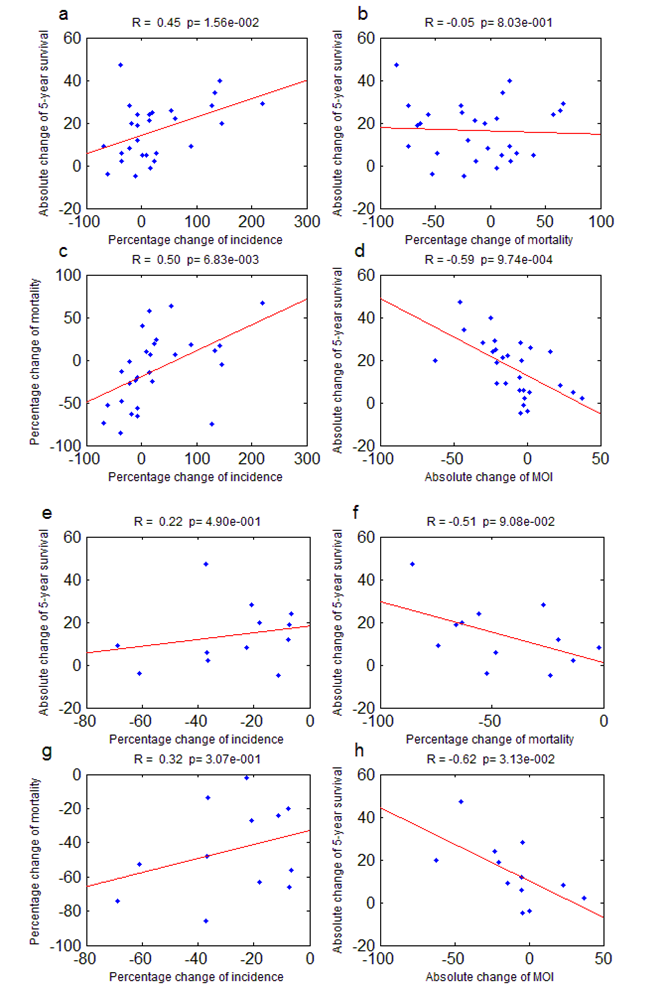

Supplement: Figure S8 — The correlation between the different measures for the male cohort from Sweden. (a) Change in mortality vs change in incidence. (b) Change in 5-year survival vs change in incidence. (c) Change in 5-year survival vs change in mortality. (d) Change in 5-year survival vs change in mortality over incidence (MOI). Pearson's correlation coefficient and its p-value are displayed on top of each panel. The change in 5-year survival is strongly linearly associated with the change in MOI. (e–h) Same as in (a–d), but including only those cancer types whose incidence decreased during the time of observation. (BMP) [file pone.0083100.s008.bmp]

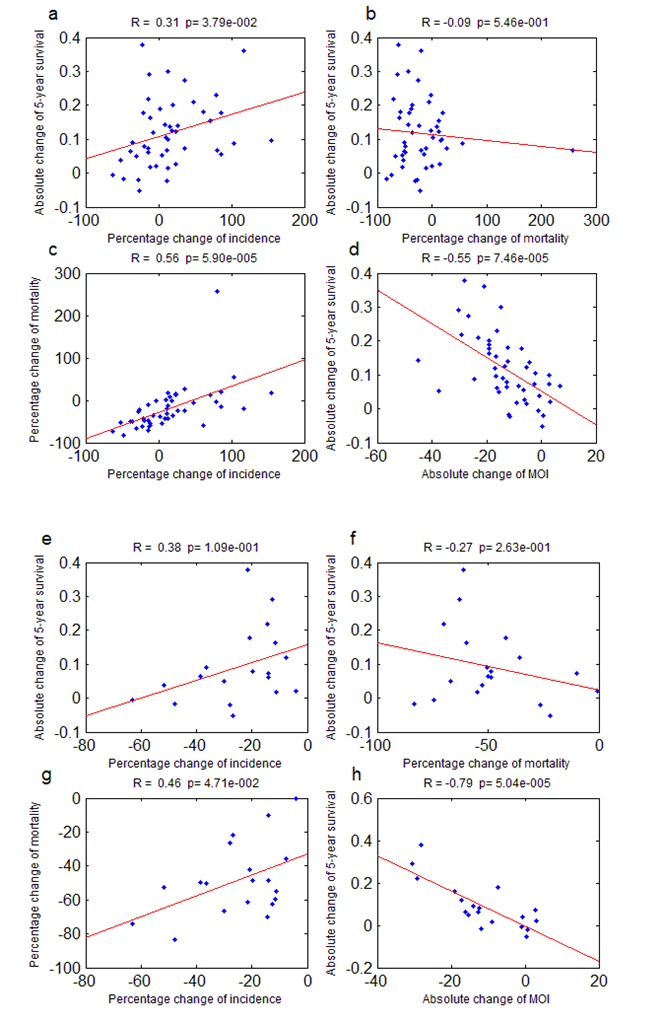

Supplement: Figure S9 — The correlation between the different measures for both genders from the US. (a) Change in mortality vs change in incidence. (b) Change in 5-year survival vs change in incidence. (c) Change in 5-year survival vs change in mortality. (d) Change in 5-year survival vs change in mortality over incidence (MOI). Pearson's correlation coefficient and its p-value are displayed on top of each panel. The change in 5-year survival is strongly linearly associated with the change in MOI. (e–h) Same as in (a–d), but including only those cancer types whose incidence decreased during the time of observation. (BMP) [file pone.0083100.s009.bmp]

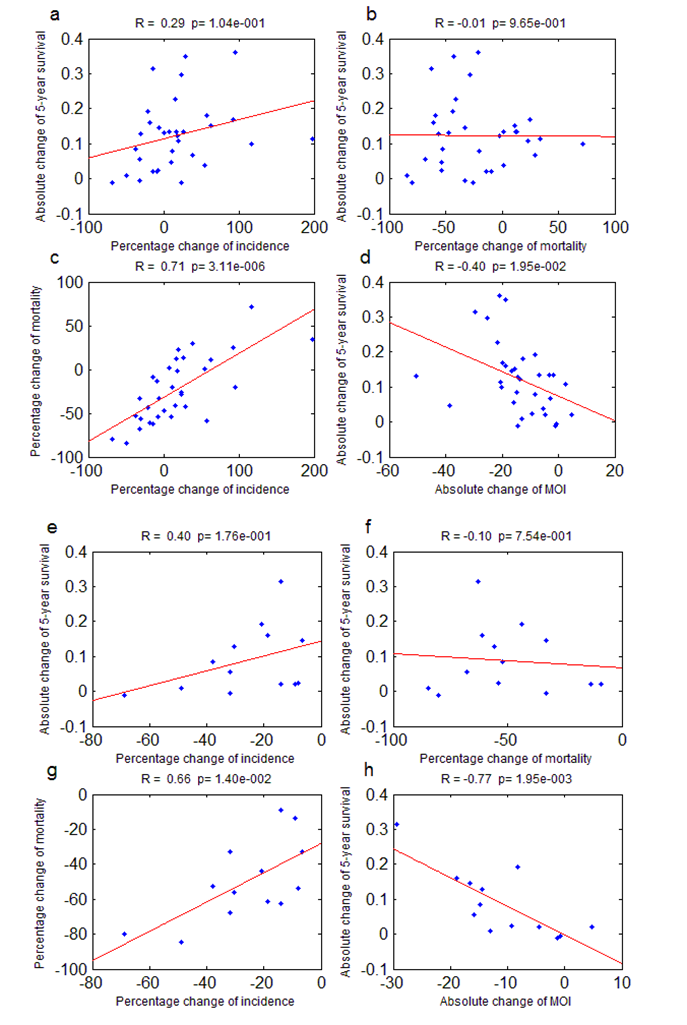

Supplement: Figure S10 — The correlation between the different measures for the male US cohort. (a) Change in mortality vs change in incidence. (b) Change in 5-year survival vs change in incidence. (c) Change in 5-year survival vs change in mortality. (d) Change in 5-year survival vs change in mortality over incidence (MOI). Pearson's correlation coefficient and its p-value are displayed on top of each panel. The change in 5-year survival is strongly linearly associated with the change in MOI. (e–h) Same as in (a–d), but including only those cancer types whose incidence decreased during the time of observation. (BMP) [file pone.0083100.s010.bmp]

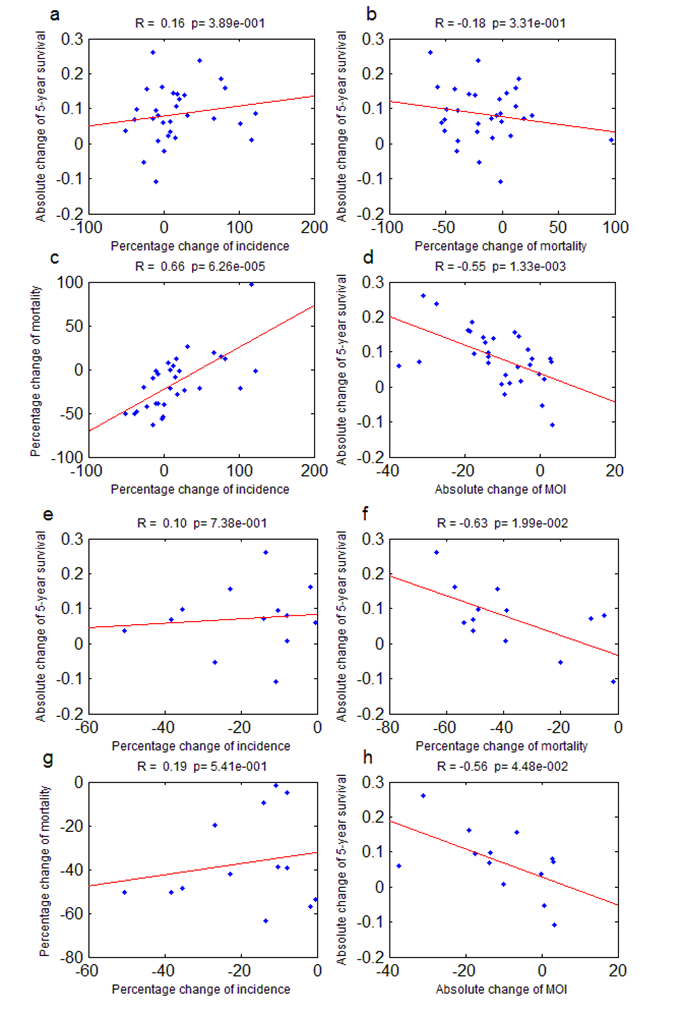

Supplement: Figure S11 — The correlation between the different measures for the female US cohort. (a) Change in mortality vs change in incidence. (b) Change in 5-year survival vs change in incidence. (c) Change in 5-year survival vs change in mortality. (d) Change in 5-year survival vs change in mortality over incidence (MOI). Pearson's correlation coefficient and its p-value are displayed on top of each panel. The change in 5-year survival is strongly linearly associated with the change in MOI. (e–h) Same as in (a–d), but including only those cancer types whose incidence decreased during the time of observation. (BMP) [file pone.0083100.s011.bmp]
